# Pneumocephalus Following Self-Inflicted Penetrating Brain Injury

**DOI:** 10.1155/2017/7878646

**Published:** 2017-09-26

**Authors:** Che-Fang Ho, Yuan-Yun Tam, Chia-Chen Wu

**Affiliations:** ^1^Department of Otorhinolaryngology-Head and Neck Surgery, Keelung Chang Gung Memorial Hospital, Keelung City, Taiwan; ^2^Department of Otorhinolaryngology-Head and Neck Surgery, Lotung Poh-Ai Hospital, Yilan County, Taiwan; ^3^Department of Otorhinolaryngology-Head and Neck Surgery, Linkou Chang Gung Memorial Hospital, Chang Gung University, Taoyuan City, Taiwan

## Abstract

**Objective:**

Pneumocephalus is a rare complication that often occurs after traumatic skull base injury, leading to morbidity and mortality.

**Material and Method:**

We present the case of a 42-year-old healthy man who injured himself when he stuck a metal stick into his left nasal cavity to relieve prolonged nasal obstruction. Immediate cerebrospinal fluid rhinorrhea and subsequent meningitis and pneumocephalus occurred later. He was presented at our hospital with fever and meningeal signs.

**Result:**

Computed tomography scans revealed left rhinosinusitis and air collection in the subarachnoid space. The patient received the conservative treatment of bed rest, intravenous hydration, head elevation, and broad-spectrum intravenous antibiotics. Pneumocephalus and meningitis resolved without any surgery, and he experienced no other sequela or complication.

**Conclusion:**

Pneumocephalus is a rare incidence and can lead to high morbidity and mortality. Prompt diagnosis and adequate treatment of pneumocephalus and meningitis proved beneficial for our patient who recovered without any complication or surgery.

## 1. Introduction

Pneumocephalus is rare and often caused by trauma; only 0.5%–1% of all episodes of head trauma result in pneumocephalus [1]. Pneumocephalus refers to the intracranial collection of gas. Cerebrospinal fluid (CSF) rhinorrhea occurs when a fistula is present between the dura and skull base, and it is also often caused by trauma [2]. In some cases, air can replace the leaked CSF on the brain surface and in the lateral ventricles, thus causing pneumocephalus. A small amount of the air could be spontaneously absorbed. In this report, we present a case of immediate CSF rhinorrhea and subsequent meningitis and pneumocephalus caused by nose picking, which resolved with conservative treatment.

## 2. Case Presentation

**Figure 1:**
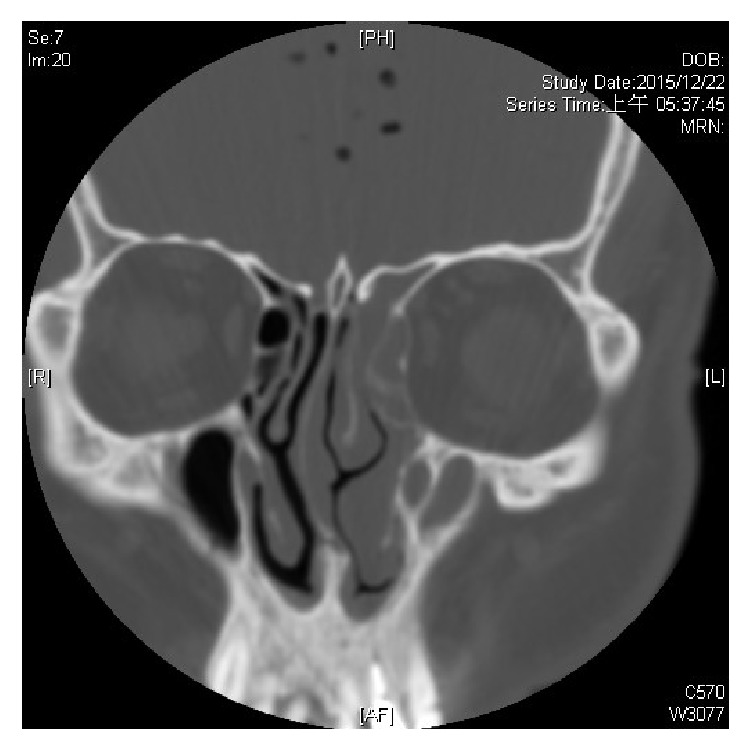
Coronal view of the CT scan reveals opacification of left ethmoid and maxillary sinus, indicating left chronic sinusitis. Note the air bubble in the left olfactory fossa, which gives us a hint of the possible site of breach in the lateral lamella of cribriform plate on patient's left side.

**Figure 2:**
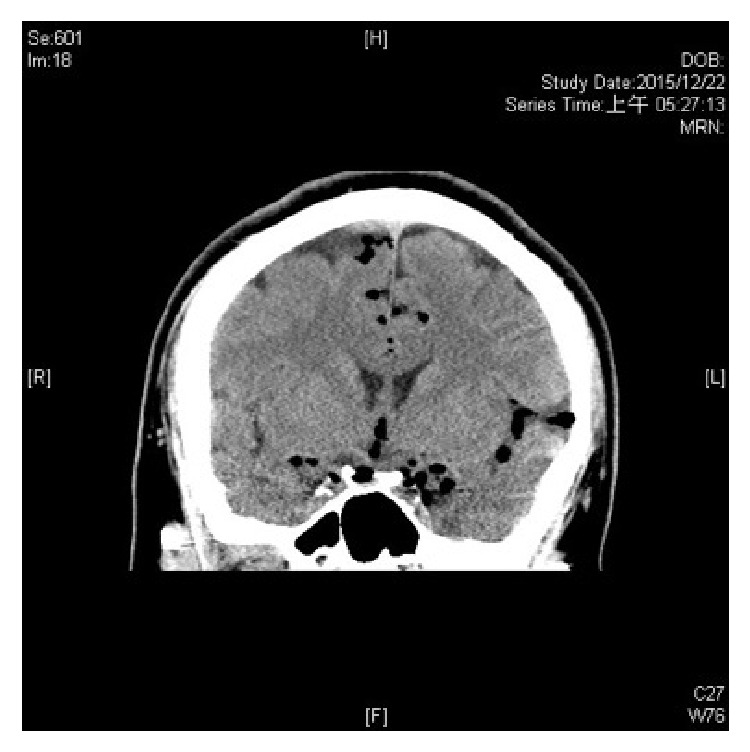
The transverse view of the CT scan shows air accumulation in the subarachnoid space indicating a pneumocephalus.

A 42-year-old healthy man with a long history of left nasal obstruction attempted to relieve his stuffy nose by inserting a 6–8 cm long metal stick into his left nostril. He reported hearing a click sound followed by bleeding from the left nasal cavity. The epistaxis stopped shortly after local compression, and the patient blew his nose to clear the residual blood clots. He then noticed a clear, watery discharge from the left nostril. Six hours later, diffuse headache developed. The watery rhinorrhea persisted for the next 2 days, and the headache became intolerable. So he visited the emergency department of our hospital. Computed tomography (CT) scan revealed left maxillary and ethmoid sinuses opacification and the possible site of breach in the lateral lamella of cribriform plate on patient's left side (Figure 1). CT also showed multiple air formation in the subarachnoid spaces, suggesting pneumocephalus (Figure 2). On examination, he had signs of meningeal irritation, including nuchal rigidity, but no other focal neurological signs or symptoms. His body temperature was 38.0°C, blood pressure was 133/68 mm Hg, pulse rate was 104 beats per minute, and respiratory rate was 20 breaths per minute. His laboratory parameters showed elevated white blood cell count (11,100/mm^3^; neutrophils, 87%, and lymphocytes, 7.2%) and C-reactive protein (CRP; 63.5 mg/L). Through sinuscopy, neither CSF leakage nor gross destruction of nasal structures could be detected. The patient was admitted and was advised bed rest for 6 days and to avoid coughing, sneezing, nose blowing, and anything strenuous. Empiric antibiotics, such as vancomycin, ceftriaxone, and metronidazole, were intravenously administered to treat meningitis. His fever subsided, and the headache and meningeal signs gradually resolved. The follow-up blood test revealed no leukocytosis, and CRP was in the reference range of 3.97 mg/L. The patient was discharged after 6 days of supportive treatment without surgical intervention.

## 3. Discussion

CSF rhinorrhea can be a complication in both sinonasal surgery and skull base surgery [3]. It might result in meningitis and pneumocephalus, which may lead to morbidity and mortality. Pneumocephalus is often caused by trauma; only 0.5%–1% of all episodes of head trauma result in pneumocephalus [1]. The accumulation of intracranial air can be acute (<72 h) or delayed (≥72 h) [4]. Until date, the definite mechanism of pneumocephalus remains debated. Two known theories exist. The first theory is the ball valve mechanism proposed by Dandy in 1926 [5]. In this theory, air is forced by positive pressure actions, such as sneezing, into the cranium through a cranial defect, which serves as a one-way valve. The second theory is the inverted-soda-bottle effect proposed by Horowitz [6], in which negative ICP results from an excessive loss of CSF through a fistula or an iatrogenic lumbar drain. Air enters the intracranial space through the fistula or drain due to negative ICP. In 1967, Markham reviewed a total of 295 cases of pneumocephalus [7]. Trauma and neoplasm were the etiological causes in 218 (73.9%) and 38 (12.9%) cases, respectively, whereas in 26 cases (8.8%), infection was the cause; in 11 cases (3.7%), pneumocephalus was secondary to some type of surgery. The most common locations for iatrogenic injury to the skull base during endoscopic sinus surgery are the lateral lamella of the cribriform, posterior fovea ethmoidalis, frontal recess, and sphenoid sinus [7].

Our case consisted with the inverted-soda-bottle effect theory [8]. The patient injured his nose with a metal stick and might have caused a fracture to the lateral lamella of the cribriform, which is the known thinnest and most vulnerable part of the skull base [9]. CSF rhinorrhea developed after he blew his nose. As the theory describes, the CSF flowed out through a dural-arachnoid tear, causing a negative ICP and enabling air to enter the cranium, thus resulting in subsequent pneumocephalus. The skull base defect also caused meningitis because of an intracranial contamination of pathogen from chronic rhinosinusitis.

Abuabara [2] reported that most CSF leaks spontaneously close within 7–10 days, and pneumocephalus is typically absorbed without any clinical manifestation [10]. The conservative treatment of a CSF leak includes head elevation, bed rest in addition to stool softeners, prophylactic antibiotics, and acetazolamide if accompanied by elevated ICP.

A standard treatment of pneumocephalus has not been established. A literature review indicated that conservative treatment resulted in a spontaneous absorption of air within 2-3 weeks in 85% of cases [10]. However, surgical decompression is the first-line treatment for tension pneumocephalus [10], which might be life-threatening. Our patient showed no signs of tension pneumocephalus either on the CT scan or clinically (i.e., agitation, delirium, altered level of consciousness, and hemodynamic changes) [11] so emergent surgical intervention was not indicated.

The CSF rhinorrhea of our case already ceased during examination, and the possible skull base defect could not be detected either on the CT or on endoscopy. Hence, surgery was not required. The value of prophylactic antibiotics in patients with CSF leakage is debatable. However, our case already had fever, a laboratory proof of systemic infection (leukocytosis and an elevated CRP level), and presented with meningitis. Therefore, the use of broad-spectrum antibiotics was reasonable. Accordingly, our patient recovered with medical treatment without any further surgery, and no other neurological sequela was observed.

## 4. Conclusion

Pneumocephalus is rare and can lead to morbidity and mortality. Pneumocephalus following self-inflected self-inflicted penetrating brain injury, as documented in our case, is extremely rare. The patient experienced an unexpected skull base injury, causing possible CSF rhinorrhea, subsequent pneumocephalus, and meningitis. However, prompt diagnosis and adequate treatment of pneumocephalus and meningitis proved beneficial for our patient who recovered without any complication or sequela.
